# Supplementary material for: Pathological Changes in Early Medieval Horses from Different Archaeological Sites in Poland
Source: Animals (Basel). 2024 Feb 1;14(3):490. doi: 10.3390/ani14030490 (PMC10854896; doi:10.3390/ani14030490)
Supplement: Supplementary file 1 [file animals-14-00490-s001.zip › table_supplementary_pathology vs_anatomy_final(2).pdf]

Table Statistics of recorded pathologies by anatomy

| Anatomical region | Anatomical element | Recorded cases | |
|---|---|---|---|
| | | (n) | % |
| Head (n=59; 31.7%) | Skull | 16 | 8.6 |
| | Teeth | 43 | 23.1 |
| Spine and tail (n=50; 26.9%) | Cervical vertebrae | 2 | 1.1 |
| | Thoracic vertebrae | 30 | 16.1 |
| | Lumbar vertebrae | 16 | 8.6 |
| | Sacral bone | 1 | 0.5 |
| | Tail | 1 | 0.5 |
| Costa (n=3; 1.6%) | Costa | 3 | 1.6 |
| Limbs (n=74; 39.8%) | Proximal elements | 15 | 8.1 |
| | Metacarpus/metatarsus | 36 | 19.4 |
| | Phalanges | 23 | 12.4 |
| ∑ | | 186 | 100.0 |
